# Silent Burdens in Disease: Fatigue and Depression in SLE

**DOI:** 10.1155/2014/790724

**Published:** 2014-01-28

**Authors:** R. Fonseca, M. Bernardes, G. Terroso, M. de Sousa, M. Figueiredo-Braga

**Affiliations:** ^1^Department of Clinical Neurosciences and Mental Health, Faculty of Medicine, University of Porto, 4200-319 Porto, Portugal; ^2^Rheumatology Department, São João Hospital, 4200-319 Porto, Portugal; ^3^IBMC/GABBA, University of Porto, 4150-180 Porto, Portugal

## Abstract

At a time when health is being recognized as more than just avoiding death, age and comorbidity are becoming increasingly important aspects of chronic disease. Systemic Lupus Erythematous (SLE) is probably one of the best paradigms of modern chronic disease, sitting at the crossroads of numerous somatic health problems, immune activation, depression, pain, and fatigue. One hundred forty-eight female participants were enrolled in the present study: 50 diagnosed with SLE, 45 with major depressive disorder (MDD), and 53 age-matched controls. Statistically significant lower scores in quality-of-life dimensions related to physical impairment were found in SLE. Patients with MDD presented significant levels of pain, reduced physical summary component (PSC), and general health scores different from healthy controls. Fatigue was reported in 90% of women with SLE and 77.8% of the MDD patients in contrast with 39.6% in the control group. Significant correlations were seen among fatigue severity, age, and educational level in SLE. From our own previous work and more recent work on the association of immune activation and depression, unexplained fatigue in SLE may signify an early sign of immune activation flare-up. The search for cytokine markers should perhaps be extended to fatigue in SLE.

## 1. Introduction

The more recent analysis of the Global Burden of Disease has identified mental disorders and musculoskeletal diseases as some of the major contributors to Years Lived in Disability (YLD) [1]. One consequence of this study that has particularly interested us has led to the question of how such global findings can be reflected in the care of the individual patient and the understanding of the complexity of comorbidity in chronic disease. Although fatigue, anxiety, and depression crosstalk with the clinical presentation and progression of Systemic Lupus Erythematosus (SLE), clinicians generally pay more attention to the somatic health problems posed by illness [2]. This can be explained in part by present-day medical education's emphasis on the biological, genomic, and statistically significant dimensions of disease.

Studies of SLE patients have shown, however, the impact of individual elements such as anxiety and depression on the course of disease as experienced by individual patients and how, collectively, such elements have an impact on health care costs [3].

SLE is also a particularly good example of how progress in the dissection of genes and molecules involved in autoimmunity continues to be disappointingly reflected in helping the individual patient [4]. In addition, SLE as a chronic inflammatory autoimmune disease with multisystem involvement, secondary to the production of autoantibodies and to the production of Type 1 interferon by innate immune cells [5, 6], represents a singularly revealing model of the crossroads that individual clinicians or clinical teams must face to deal adequately with an individual patient.

Presently, clinical evaluation of disease relies mostly upon objective criteria, contemplating clinical features whose significance may nevertheless escape the patient. Particular symptoms such as pain and fatigue can, on the contrary, be experienced and reported exclusively by the patient, escaping regular clinical assessment. We would like to identify such individual burdens as silent burdens of disease.

Eventually, the disrupted immunological tolerance seen in SLE patients results in immune complex deposition that ends in permanent damage, most frequently affecting the musculoskeletal, cutaneous, renal, central nervous, and gastrointestinal systems [7]. The diversity of the resulting clinical manifestations led to the establishment of consensual, valuable, sensitive, and specific diagnostic criteria: the American College of Rheumatology (ACR) Criteria for Classification of Systemic Lupus Erythematosus. Although not formally contemplated in these criteria, fatigue is the most common symptom in SLE, affecting 67% to 90% of the patients, even in mild disease presentations [8–10]. Patients often describe their fatigue as debilitating, causing a severe impact on personal, family, and social functioning [10]. Resulting from a complex interplay of biological, behavioral, and psychological factors, fatigue appears to have a privileged association with depressive symptoms, independently of genetic background [11]. Indeed, fatigue cannot be seen as a purely physical sign, as it is also a common symptom in depressive disorder [12].

Anxiety and depression are highly prevalent among patients with lupus, thought to represent central nervous system involvement [13] or immune dysfunction manifestation [14, 15], or denoting the emotional burden of the disease [16, 17]. Recently, a link has been established among neurotransmitter dysfunction, immune activation (lymphocyte abnormalities and cytokine expression), and major depression [18, 19]. In this paper, we consider fatigue and depression as examples of silent burdens of disease, in a cross-sectional study of three groups of participants: patients with SLE, patients with major depressive disorder, and age- and sex-matched controls.

The principal objective of the work was twofold: to highlight the importance of single patient derived symptoms over the course of the disease and to stress the importance of SLE as a modern model chronic disease as important as cancer, if not more so. This importance is derived from the multiple effects resulting from the challenge of crosstalk between the immunological system and other systems, in the absence of infection.

## 2. Study Participants and Methods

One hundred forty-eight female participants were enrolled in the present study, 50 of whom were patients diagnosed with SLE, recruited from the rheumatology outpatient clinic of the São João Hospital, EPE. All SLE patients fulfilled the ACR diagnostic criteria. Only adult patients (>18 years old) without diagnosed psychiatric comorbidities were selected. All patients attended routine visits at the hospital and completed regular clinic and laboratorial assessments.

Patients with lupus were compared to one group of 53 healthy age-matched subjects and a group of 45 patients with major depressive disorder (MDD), followed by one of the authors (mmfb) in her private psychiatric clinic. Psychiatric patients were diagnosed by a psychiatrist according to the Diagnostic and Statistical Manual of Mental Disorders (DSM-IV-TR) [20]. The study was submitted and approved by the Ethical Committee of the São João Hospital (EPE). Participants received oral and written information about the study's goals, methods, expected benefits, and discomfort, after which they gave their oral and written informed consent. The confidentiality and privacy of the collected data were guaranteed during the data collection and analysis stages according to the Declaration of Helsinki.

The sociodemographic characterization of the populations studied is shown in Table 1.

## 3. Data Collection

Following a cross-sectional approach, psychosocial data were collected between September 2012 and June 2013. A first contact established the willingness of the patients to participate. After a first verbal consent was obtained, written informed consent was mailed and retrieved from all participants before the study began. Recruited patients and controls were subsequently interviewed by phone by a trained interviewer (RF). The literature corroborates phone interviews as valid and precise tools for psychological data collection [21–25].

Participants' sociodemographic data included age, educational level, employment status (active/nonactive), and marital status. Laboratorial and SLE standardized clinical evaluation were obtained exclusively in the SLE patients through the clinical records.

## 4. Instruments

### 4.1. Psychosocial Evaluation

Psychological variables were obtained through a battery test: the Fatigue Severity Scale (FSS), the Hospital Anxiety and Depression Scale (HADS), the Pittsburgh Sleep Quality Index (PSQI), and the Short Form-36 Health Survey (SF-36v2).

Self-reported fatigue was evaluated through the short version of the FSS [26]. This unidimensional nine-item Likert scale was designed to assess fatigue in chronic medical and rheumatologic conditions and is recommended as the instrument of choice for research purposes in studies involving patients diagnosed with SLE [27]. The Portuguese version of the FSS used in the present work has been validated for SLE patients [28].

The FSS demonstrates good internal consistency (Cronbach's *α* = 0.89 in SLE patients), test-retest reliability (0.84), and construct and discriminative validity and is sensitive to change. Each question is scored from 1 to 7, and a final score is obtained from the mean of all scored items. Higher scores reveal increasing severity of fatigue. Presence of clinical levels of fatigue was defined by a FSS score >3, as proposed by Krupp and collaborators [26]. The use of FSS assessment in an SLE population by telephone interview has been established [29].

The Hospital Anxiety and Depression Scale (HADS) [30] is used to assess depressive and anxiety symptoms. This questionnaire contains 14 items, scored from 0 to 3, to achieve a total of 0 to 21. It is divided into two sets of seven questions aiming to detect, respectively, depressive and anxiety states. Scores exceeding 8 points indicate possible mood disorder, and 10 points delimit pathological situations. This instrument does not contain items focused in physical indicators of psychological distress or somatic complaints, which improves its sensitivity to anxiety and depression in physically ill individuals. Factor analysis confirmed the bidimensionality of the scale and the correlation between the anxiety and depression subscales. Both subscales showed suitable internal consistency (Mean Cronbach's alpha = 0.83 for HADS-A and 0.82 for HADS-D) [31]. Telephone-administered mode has been described for the HADS, maintaining similar psychometric properties [32].

Participants were also screened for sleep quality through the Pittsburgh Sleep Quality Index (PSQI). A weighted global score of 0 to 21, reflecting a four-week time interval, is yielded from the seven components subjectively evaluated: sleep latency, sleep disturbances, sleep duration, sleep quality, sleep efficiency, use of sleep medications, and daytime dysfunction. This instrument has good psychometric properties, with high homogeneity, reliability (Cronbach's alpha = 0.83), and validity [33, 34]. Poor sleepers are identified by a PSQI score >5 [33]. When used in a telephone interview, the PSQI presented an adequate internal consistency and proved to be a reliable mode for sleep quality assessment [35, 36].

The Short-Form 36 Health Survey Version 2.0 (SF-36v2) was elected to assess health-related quality of life (HRQoL) [37]. This scale comprises 36 self-rated questions, reproducing eight domains: physical functioning (PF), role limitations due to physical problems (RP), bodily pain (BP), general health (GH), vitality (VT), social functioning (SF), role limitations due to emotional problems (RE), and mental health (MH). These domains can be individually evaluated or summarized in the physical (PSC) and mental (MSC) summary components. A gradual score ranging from 0 to 100 in each area reflects enhanced quality of life. The SF-36 can be either self-administered or administered by a trained interviewer, either in person or by telephone [38, 39]. The SF-36-v2 is the most widely used questionnaire to assess quality-of-life-related outcomes in SLE patients, providing the additional possibility of comparing the obtained results with those of other healthy or patient populations [40].The Cronbach's alpha for studies enrolling SLE patients was recently computed from the available literature (Cronbach's *α* = 0.71–0.95) [41].

### 4.2. Clinical SLE Indexes

Disease activity was estimated with the Systemic Lupus Erythematosus Disease Activity Index (SLEDAI) [42]. This physician-rated instrument reports 16 clinical and eight laboratorial descriptors appraised during the previous 10 or 30 days. The SLEDAI score retrieved during the last (most recent) patient visit was used. Patients were grouped into four categories, according to SLEDAI total score, representing increasing activity of the disease: no activity (0), mild activity (1–5), moderate activity (6–10), high activity (11–19), and very high activity (20) [43]. A score of 6 or more was considered as clinically active disease.

The Systemic Lupus International Collaborating Clinics/American College of Rheumatology (SLICC/ACR) damage index [44] was used to compile irreversible impairment in all SLE patients. This index reflects accumulated damage continuously present in a six-month period and targets 12 distinct organs/systems.

### 4.3. Laboratory Evaluation

Laboratorial evaluation included in the patient's clinical records was used to determine immune activation, inflammatory status, antibody profile, and vitamin D quantifications. Immune activation was determined by autoantibodies detection: antidouble stranded DNA, nucleosome, Smith, Sjogren Syndrome A, Sjogren Syndrome B, histones, cardiolipin (IgG and IgM isotypes), *β*2-glycoprotein I (IgG and IgM isotypes), antiribonucleoproteins, and Ribosomal P substance. The C-Reactive Protein (CRP), inactive C1, C1q, C3c, C4, and CH50 complement fractions and Erythrocyte Sedimentation Rate (ESR) determinations were also considered. Blood and serological measurements were performed according to standardized methods in the hospital's laboratory, and standardized cutoff levels were established. Only two laboratory tests correlate with fatigue: CH50 and anti-ds-DNA determinations (data not shown).

### 4.4. Statistical Analysis

Statistical analysis was performed using the Statistical Package for Social Science 18.0 (SPSS).

A descriptive analysis of the obtained results was performed, and the data were expressed as frequencies (%), minimums, maximums, means, and standard deviations.

Analysis of variance (ANOVA) was used to investigate differences between groups, and Pearson Chi-square test was conducted for categorical variables. Post-hoc Tukey test was performed to provide a stratified comparison between groups, when necessary. For correlation analysis, the Spearman's coefficient was computed. Confidence intervals of 95% and a significance level of 0.05 were adopted.

## 5. Results

### 5.1. Fatigue Assessment: Significant Associations with Age, Psychological Suffering, and Educational Level (Tables 1, 2, and 3)

The sociodemographic characterization of the three groups showed that all participants presented similar age, with a mean value of 44.5 years and similar marital status. Regarding employment status and education, however, lower scores were observed in the SLE group, with statistical significance when compared to depressed and control women (Table 1).

Fatigue was not an exclusive burden of SLE. It was reported in 90% of women with SLE and 77.8% of the female MDD patients, in contrast with 39.6% in the control group.

The global score of FSS revealed similar fatigue severity in SLE and MDD patients (5.2 ± 1.3 and 4.5 ± 1.4, resp.), which is significantly higher than the scores found in the control group (3.2 ± 1.8, *P* = 0.0001) (Table 2).

The result of the search for significant correlations between fatigue and markers of psychological suffering is detailed as a correlational analysis in Table 3.

Significant correlations were detected between fatigue severity, age, and education exclusively in patients with SLE. Higher fatigue was associated with older age, possibly reflecting the cumulative physical impairment of disease and aging. Lower education was seen to be related to higher fatigue scores.

Significant correlations between anxiety and fatigue were detected in SLE and control population (*r* = 0.542 and *r* = 0.397, resp.).

Mental health scores, vitality, and depressive symptoms presented significant associations with fatigue severity, regardless of participant group (Table 3).

In the SLE group of women, education correlated significantly with anxiety (HADS-A: *r* = 0.313,  *P* = 0.027), depressive symptoms (HADS D *r* = 0.452,  *P* = 0.001), and bodily pain (*r* = 0.527,  *P* < 0.005).

### 5.2. Weight, Sleep, and Physical Activity (Table 4)

In order to clarify classically reported associations among fatigue, excessive weight, sleep abnormalities, and physical activity, participants were subjected to anthropometric, sleep quality, and health-related behavior evaluations.

In our study, disturbed sleep quality affected the three groups, although higher global PSQI scores (*P* = 0.007), representing poorer sleep quality, were detected in SLE and MDD patients.

The presence of obesity and preobesity was equally distributed across the studied sample, and BMI scores were similar in all the groups. Evaluation of health-related behaviors revealed that physical activity and alcohol consumption were similar in the three groups. Smoking habits, on the contrary, were more prevalent in psychiatric patients (Table 4).

### 5.3. Fatigue and Quality-of-Life Dimensions: Physical Summary Components, Pain, and General Health (Table 5)

Fatigue has been extensively associated with poorer health-related quality of life in SLE patients. Accordingly, we detected statistically significant lower scores in quality-of-life dimensions related to physical impairment in SLE patients; results in physical summary components, bodily pain and general health were statistically significantly different in the SLE group when compared to the MDD group and control subjects. MDD patients, however, presented significant levels of pain, reduced PSC, and general health scores different from healthy controls.

Other dimensions expressed in mental summary components (social functioning, mental health, and role limitations due to emotional problems), while showing significant impairment in SLE patients, presented lower scores in the MDD group when compared to the control population (Table 5).

### 5.4. Fatigue, Anxiety, and Depression (Table 6)

Loss of energy or exhaustion can characterize fatigue and also be regarded as a depressive symptom. In the present study, measures of depression were obtained using HADS depression subscale in the three groups of participants, and they revealed the occurrence of similar depressive symptoms in SLE patients and MDD patients (6.7 ± 5.0 and 8.4 ± 4.0, resp.), which are significantly higher than in the normal population. Anxiety presented higher scores in MDD patients (11.4 ± 3.7), which are statistically significantly different from the SLE and control groups.

### 5.5. Lack of Correlations between Clinical SLE Evaluation and Severity of Fatigue (Table 7)

Clinical indexes used to assess disease activity and damage in SLE patients did not present significant associations with fatigue severity, failing to translate subjective patients' complaints into clinical standardized evaluation.

## 6. Discussion

The spectacular progress in our understanding of the molecular and genetic basis of disease is transforming clinical practice and the nature of patient-physician interaction. Indeed, distracted by the panoply of biological markers at his/her disposal, the clinician may miss subjective symptoms of relevance to the development of disease. Fatigue, anxiety, and depression fall in the category of subjective symptoms that may escape the attention of clinicians *and* patients. Because of their impact in the course of a chronic disease such as SLE, such symptoms will become of increasing clinical and social value as the concept of health itself changes. As emphasized by others, “health is about more than avoiding death” [1].

As shown by the results of the present study, some correlations of fatigue, for example, with sleep quality were common to all groups examined, namely, SLE, MDD, and the control group. A correlation of fatigue with anxiety was also seen within the control group.

The presence of obesity and BMI scores was similar in all groups just as alcohol consumption and physical activity. Smoking habits were more prevalent in the psychiatric group.

We would like to highlight two observations made exclusively in SLE: the correlation with age and education and the correlation with depression (not exclusively seen in SLE).

### 6.1. Fatigue and Depression

In a recent analysis of fatigue in monozygotic and dizygotic twins, chronic fatigue and psychological distress were strongly associated without evidence of genetic covariation, implying, according to the authors, that the “association is environmental” [11]. In an earlier large analysis of a World Health Organization longitudinal study of fatigue and depression, Skapinakis et al. concluded that unexplained fatigue and depression might act as independent factors of each other [45].

However, Palagini et al., in a review based on the search of SLE and depression as key-words in several major databases, concluded that to date, the relationship between depression and SLE disease activity appears controversial, stressing the need for identification of SLE-specific biomarkers of depression. Methodological limitations are present in the available literature, and the standardization of methodologies should be considered a high priority in SLE research [46].

In the present cross-sectional study, we confirmed the existence of a link between fatigue and depression in the patients studied. From our own previous work [14] and more recent work on the association of immune activation and depression, unexplained fatigue may signify an early alert signal of a flare-up of immune activation [18, 19], and the search for cytokine markers in SLE [47] should perhaps be extended to fatigue in SLE.

### 6.2. Fatigue and Education Level

Comparison of educational levels can be done only between the SLE and control groups. The link between education level and fatigue strengthens the point made by others about the importance of the role of health professionals, including nurses, in explaining the disease to patients [48]. Patients with lower education levels challenge clinicians and other health professionals' ability to gather and share important information. Reduced educational achievement may affect a patient's ability to understand, and, in addition, to seek appropriate clarification of doubts regarding the disease, the treatment, or its expected outcomes [49]. Lack of knowledge can comprehensibly add more anxiety and suffering to the difficulty of living with a disease with uncertain evolution and unpredictable flares.

## 7. Conclusion

Giving the attention that fatigue and depression may deserve as silent burdens of disease in SLE, we may be preventing a deleterious progression of a disease and, thus, diminishing the costs recently estimated in Sweden, where a total of 339 patients with the mean age of 55 years were analyzed. The mean Health Related Quality of Life (HRQoL) measured through the five-item EQ-5D instrument was 0.64, and total costs were estimated at €22,594 (direct costs €7,818; indirect costs €14,776). Disease activity, fatigue, and corticosteroid doses had a statistically significant impact on costs and HRQoL. This study demonstrates that Swedish patients with SLE have low HRQoL and incur high societal costs that are both associated with and most likely driven by disease activity, fatigue, and corticosteroid use [3].

The objective measure of costs has, like molecular and genetic progress, become a carefully “listened to” burden of health care and chronic disease.

We wish to conclude by returning to our starting reference to the Global Burden of Disease study, referencing one of their interpretations: *“Prevalences of the most common causes of YLDs such as mental and behavioural disorders and musculoskeletal disorders have not decreased. Health systems will need to address the needs of the rising numbers of individuals with a range of disorders that largely cause disability but not mortality”*—such as SLE [1].

The health system will work well proportionally to the attention and time clinicians can give to one patient. We hope this study of SLE as a model of all modern chronic diseases, with all its limitations as a cross-sectional study and its small numbers, will nevertheless help clinicians and patients to become aware of the importance of the weight of their silent burden in the global burden of modern disease, where *“health is about more than avoiding death” *[1].

Identifying silent burdens in SLE is essential at all times to the care and follow-up of disease progression.

## Figures and Tables

**Table 1:** Sociodemographic characterization.

	Total *n* = 148	SLE (1) *n* = 50	Depression (2) n = 45	Controls (3) n = 53	P	Post hoc analysis
Age (years)^a^	44.5 (11.6)	44.1 (10.1)	41.7 (13.7)	47.2 (11.8)	0.108^c^	
Education (years)^a^	10.7 (4.8)	9.1 (3.9)	13.0 (4.2)	10.9 (5.4)	0.001^c^	2 > 1, 3
Marital status^b^						
Single	26 (17.6)	8 (16.0)	10 (22.2)	8 (15.1)		
Married	101 (68.2)	38 (76.0)	25 (55.6)	38 (71.7)		
Divorced	15 (10.1)	2 (4.0)	9 (20.0)	4 (7.5)	0.254^d^	
Widow	4 (2.7)	1 (2.0)	1 (2.2)	2 (3.6)		
Common-law marriage	2 (1.4)	1 (2.0)	0 (0.0)	1 (1.9)		
Employment status^b^						
Active	86 (58.1)	15 (30.0)	27 (60.0)	44 (83.0)	0.000^d^	
Nonactive	62 (41.9)	35 (70.0)	18 (40.0)	9 (17.0)		

^a^Mean (standard deviation), ^b^ *n* (%), ^c^ANOVA, ^d^Chi-square test.

**Table 2:** Fatigue assessment.

	Total *n* = 148	SLE (1) *n* = 50	Depression (2) *n* = 45	Controls (3) *n* = 53	*P*	Post hoc analysis
FSS global score^a,b^	4.3 (1.7)	5.2 (1.3)	4.5 (1.4)	3.2 (1.8)	0.000^d^	2, 1 > 3
	1.0–7.0	1.6–7.0	1.7–7.0	1.0–10.4		
Fatigue severity level^c^						
Nonclinical	36 (24.3)	5 (10.0)	6 (13.3)	25 (47.2)	0.000^e^	
Clinical	101 (68.2)	45 (90.0)	35 (77.8)	21 (39.6)		
Missing	11 (7.4)		4 (8.9)	7 (13.2)		

^a^Mean (standard deviation); ^b^minimum–maximum; ^c^ *n* (%); ^d^ANOVA; ^e^Chi-square test.

**Table 3:** Correlations (Pearson) between fatigue and psychosocial and anthropometric characteristics, quality of life, and sleep quality.

	SLE (1) *n* = 50	Depression (2) *n* = 45	Controls (3) *n* = 53
Age (years)	0.489**	−0.061	0.243
Education (years)	−0.476**	−0.043	−0.294
HADS			
Anxiety	0.542**	0.202	0.397**
Depression	0.576**	0.402*	0.410**
SF-36			
PSC	−0.717**	−0.351*	−0.466**
Physical functioning	−0.648**	−0.103	−0.318*
Role limitations due to physical health problems	−0.699**	−0.393*	−0.302*
Social functioning	−0.354*	−0.389*	−0.226
Mental health	−0.464**	−0.416**	−0.435**
Role limitations due to emotional problems	−0.315*	−0.249	−0.274
Vitality	−0.677**	−0.323*	−0.660**
Bodily pain	−0.563**	−0.450**	−0.287
General health	−0.617**	−0.418**	−0.580**
PSQI global score	0.401**	0.334*	0.425**
BMI (kg/m^2^)	0.135	−0.82	−0.301*

*P < 0.05, **P < 0.01; PSC: Physical summary component; BMI: body mass index.

**Table 4:** Anthropometric characterization, health-related behaviors, and sleep quality.

	Total *n* = 148	SLE (1) *n* = 50	Depression (2) *n* = 45	Controls (3) *n* = 53	*P*	Post hoc analysis
BMI (kg/m^2^)^a^	24.9 (5.5)	24.8 (4.1)	25.7 (7.8)	24.3 (4.0)	0.466^c^	
BMI categories^b^						
Underweight	7 (4.7)	1 (2.0)	3 (6.7)	3 (5.7)	0.701^d^	
Normal range	85 ( 57.4)	31 (62.0)	24 (53.3)	30 (56.6)		
Preobesity	32 (21.6)	13 (26.0)	8 (17.8)	11 (20.8)		
Obesity	18 (12.2)	5 (10.0)	8 (17.8)	5 (9.4)		
Missing	6 (4.1)	0 (0.0)	2 (4.4)	4 (7.5)		
Smoking habits^b^						
Smokers	34 (23.0)	10 (20.0)	18 (40.0)	6 (11.3)	0.003^d^	
Nonsmokers	114 (77.0)	40 (80.0)	27 (60.0)	47 (88.7)		
Alcohol consumption^b^						
Yes	13 (8.8)	3 (6.0)	2 (4.4)	8 (15.1)	0.124^d^	
No	135 (91.2)	47 (94.0)	43 (95.6)	45 (84.9)		
Physical activity^b^						
Yes	63 (42.6)	18 (36.0)	18 (40.0)	27 (50.9)	0.283^d^	
No	85 (57.4)	32 (64.0)	27 (60.0)	26 (49.1)		

Sleep						
PSQI global score^a^	10.3 (3.8)	10.9 (4.0)	11.3 (3.7)	9.0 (3.4)	0.007^c^	2, 1 > 3
Quality of sleep^b^						
Good sleepers	16 (10.8)	3 (6.0)	3 (6.7)	10 (18.9)	0.140^d^	
Poor sleepers	126 (85.1)	46 (92.0)	39 (86.7)	41 (77.4)		
Missing	6 (4.1)	1 (2.0)	3 (6.7)	2 (3.8)		

^a^Mean (standard deviation), ^b^ *n* (%), ^c^ANOVA, ^d^Chi-square test; BMI: body mass index.

**Table 5:** Health-related quality of life.

	Total *n* = 148	SLE (1) *n* = 50	Depression (2) *n* = 45	Controls (3) *n* = 53	*P*	Post hoc analysis
SF-36						
Physical functioning^a^	71.8 (26.7)	55.8 (30.5)	75.9 (22.2)	84.3 (16.7)	0.000^b^	1 < 2, 3
Role limitations due to physical health problems^a^	62.5 (30.8)	44.5 (32.6)	59.8 (23.5)	81.3 (22.4)	0.000^b^	1 < 2 < 3
Social functioning^a^	63.0 (28.6)	61.5 (31.0)	46.6 (22.5)	78.1 (22.5)	0.000^b^	2 < 1 < 3
Mental health^a^	56.8 (22.1)	55.8 (20.2)	43.0 (17.5)	69.3 (20.2)	0.000^b^	2 < 1 < 3
Role limitations due to emotional problems^a^	70.1 (27.9)	68.7 (29.0)	52.4 (22.9)	86.5 (20.5)	0.000^b^	2 < 1 < 3
Vitality^a^	45.6 (22.9)	34.9 (23.1)	39.4 (16.8)	70.0 (18.8)	0.000^b^	1, 2 < 3
Bodily pain^a^	60.7 (31.6)	43.7 (34.6)	64.5 (26.1)	73.7 (25.5)	0.000^b^	1 < 2, 3
General health^a^	49.8 (23.7)	31.3 (18.5)	56.2 (21.2)	61.9 (19.2)	0.000^b^	1 < 2, 3
PSC^a^	0.0 (1.0)	−0.8 (0.9)	0.4 (0.7)	0.5 (0.6)	0.000^b^	1 < 2, 3
MSC^a^	0.0 (1.0)	0.1 (0.9)	−0.8 (0.8)	0.6 (0.8)	0.000^b^	2 < 3 < 1

^a^Mean (standard deviation); ^b^ANOVA; PSC: physical summary component; MSC: mental summary component.

**Table 6:** Depression and anxiety symptoms.

	Total *n* = 148	SLE (1) *n* = 50	Depression (2) *n* = 45	Controls (3) *n* = 53	*P*	Post hoc analysis
HADS-D^a^	6.4 (4.5)	6.7 (5.0)	8.4 (4.0)	4.5 (3.5)	0.000^b^	2, 1 > 3
HADS-A^a^	8.8 (4.6)	8.5 (4.8)	11.4 (3.7)	6.8 (4.1)	0.000^b^	2 > 1, 3

^a^Mean (standard deviation); ^b^ANOVA.

**Table 7:** Correlation (Pearson) between fatigue and disease-related markers in SLE patients.

	FSS
SLEDAI	−100
SLICC	0.043
